# Embolism of the Pulmonary Artery in a Horse1Read before the fifteenth annual meeting of the Iowa State Veterinary Medical Association, Cedar Rapids, Iowa, January 14, 15, 1903.

**Published:** 1903-02

**Authors:** J. W. Scott

**Affiliations:** Manchester, Iowa


### EMBOLISM OF THE PULMONARY ARTERY IN A HORSE.[1]

By J. W. Scott, V.S.,
MANCHESTER, IOWA.

I WILL report to you a case which I diagnosed to be embolism of the pulmonary artery, but which has not been proven by post-mortem, as the animal (a bay mare about eight years old) is still alive and in the same condition as when I saw her last August. The early history of the case, which I obtained from the owner, showed that the animal had been healthy until some time during the late winter or early spring of 1902, when she commenced to show symptoms of weakness and dyspnœa when at work or moving rapidly. These symptoms were not apparent if the animal was allowed to stand. Her appetite was normal, and all the organs of excretion and secretion were normal. Her condition was attributed to the fact that she was heavy with foal, but this was disproved when parturition occurred. Her condition remained unchanged and she was kept in the pasture until an opportunity came to dispose of her.

Her owner at the time of my examination got her in a trade some two weeks prior, and he informed me that as he drove out of the

[1] Read before the fifteenth annual meeting of the Iowa State Veterinary Medical Association, Cedar Rapids, Iowa, January 14, 15, 1903.

town toward his home, which was about four miles distant, the animal showed symptoms of falling. These symptoms were speedily developed into fact, for before he could unhitch from his vehicle the animal was floundering on the ground fighting for breath, and was, as he supposed, in her death agony, from which it took about fifteen minutes to recover sufficiently to regain her feet. After resting for an hour he rode her slowly to his home and turned her to pasture, where she remained without further incident until the day I was called in to examine her.

Examination conducted in the field revealed no indication of disease, the pulse, respiration, and temperature all being normal. She was then haltered and led at a sharp trot for about one hundred yards, which occasioned some excitement, the breathing being rapid and tumultuous. We then harnessed her, being careful to select a good-fitting collar, which could in no way interfere with breathing or the free passage of blood in the jugular veins, and hitched her with another animal to an empty wagon. We started at a slow pace toward the road, which was not more than sixty yards distant. By the time we reached the gate she commenced to stagger and the driver stopped. She was quite excited, the mucous membranes were cyanotic and the breathing stertorous. The submaxillary artery was full, but pulsation was feeble. She recovered her composure very quickly. I then instructed the driver to go ahead and keep going until I asked him to stop. After going about one hundred yards, I judged from the character of her breathing, which was loud and hoarse, and the excited manner in which she threw her head from side to side, that she had about reached the limit of exertion to which she could be safely subjected, so we stopped, and, with her struggling and surging and dashing the head wildly about, we managed to get her unhitched from the wagon, but before we could remove the harness she fell to the ground, gasping for breath and giving evidence of the greatest distress. This agitated condition was of such long duration and of such severity that recovery seemed doubtful, and her efforts were so violent that I could not make any examination at this time; but, as the more furious symptoms subsided, I approached and felt for her pulse. The artery was full but motionless, the mouth and nasal membranes of a leaden color, and circulation was to all appearances completely stagnated. I placed my finger on the jugular and made an unsuccessful effort to raise the vessel. It was almost half an hour before she was able to regain a standing posture, and was not wholly recovered when I came away about one hour later.

What pathological state would be most likely to call forth these symptoms? That the trouble was one of circulation I felt well assured, and that it was thrombosis or embolism of some important vessel appeared to be the only rational conclusion. After mature deliberation, I pronounced it to be embolism of the pulmonary artery, and will give my reasons for believing that this diagnosis was correct. The common aorta and the pulmonary artery are the only vessels in the body which carry the whole volume of the blood. The latter leaves the right ventricle and proceeds to the upper level of the auricle, then backward a short distance, the entire length of the vessel being four or five inches. Here it divides into right and left branches, each of which conveys the blood to its respective lung. It is now returned to the left heart by the pulmonary veins, and the whole volume is forced by the contraction of the left ventricle through the common aorta. Occlusion of either of these vessels would cause a complete stagnation of the whole circulatory system, and would, consequently occasion just such symptoms as those I have described, and, if continued for any length of time, death would be the result. Therefore, occlusion could not have been complete in this case. Why should those symptoms be seen only when the animal is subjected to exertion? This is a question to which a conjectural answer must be given, and in answering I make use of a theory which explains this, also why it is most likely that the impediment was in the pulmonary artery. I will first call your attention to the difference between a thrombus and an embolus. A thrombus is an obstruction to a vessel by a clot which is adherent to the vessel's wall, and consequently stationary. An embolus is a clot floating in the blood stream and may not occasion any great hinderance to the blood flow unless forced along into the narrow part of the vessel, where it may completely plug it. Now, it is evident that if the difficulty were due to thrombosis, the distressing symptoms would have been constant, as any obstruction remaining at the site of formation would interfere with the blood flow to almost the same extent during the period of rest that it would when at work. This is not the case in embolic obstruction, as the symptoms are aggravated when the clot reaches a narrow part of the vessel. In the case under consideration, when the animal is exercising the blood moves in the aorta with more force, and as a consequence the obstructing mass is pushed along to the bifurcation of the artery and lodges there, producing obstruction and rendering the whole circulatory system inactive. During quiescence of the animal the embolus becomes lodged in the widest part of the dilated artery and equilibrium is restored.

But what distinguishing symptoms would be presented if the obstruction were in the systemic aorta? The answer must be conjectural and is based upon our knowledge of the anatomy of the heart. The walls of the left ventricle are of about three times the thickness of the right, and have a contractile power proportionately greater, which drives the blood with such force through the aorta that were an embolus contained therein it would be forced to the juncture of the posterior and the anterior aorta, from which place it could not recede, and the symptoms would be continuous until the heart, now distended with blood and wholly powerless to urge it past the obstruction, would cease its efforts, death occurring in a few minutes after the first indication of distress.

### Discussion.

Dr. Heck said he had a case which resembled this somewhat, and that he made a diagnosis of spasm of the glottis.

Dr. Walrod said he thought Dr. Scott's case might be one of arterio-sclerosis of the large arteries.

Dr. Repp said he would expect to find at autopsy either thrombosis alone, or aneurism combined with thrombosis, in one of the large arteries near the heart. The appearance of the trouble on exercise could be explained by the fact that the demand upon the circulation was greatest at such times, while when the animal was at rest, the demand could be supplied notwithstanding the obstruction.

Dr. Simpson mentioned two cases in horses in which there were heart murmurs, but he did not ascertain their exact nature.

